# Newborn haemorrhagic disorders: about 30 cases

**DOI:** 10.11604/pamj.2017.28.150.13159

**Published:** 2017-10-18

**Authors:** Brahim El Hasbaoui, Lamia Karboubi, Badr Sououd Benjelloun

**Affiliations:** 1 Paediatric Medical Emergency Department, Children’s Hospital, Faculty of Medicine and Pharmacy, University Mohammed V, Rabat, Morocco

**Keywords:** New-bornhaemorrhagic disease, vitamin K, breastfeeding

## Abstract

The haemorrhagic disorders are particularly frequent in neonatal period. Their causes are varied and their knowledge is capital for their good management. Our purpose was to describe the epidemiological, diagnostic, and common causes of new-bornhaemorrhagic syndrome in paediatric emergency medical department of the Rabat Children's Hospital. We conducted a descriptive study from December 2015 to April 2016, about new-borns admitted to medical emergencies for haemorrhagic syndrome defined by bleeding, exteriorized or not, whatever its importance, severity, causes and the associated clinical and biological disorders. Between December 2015 and April 2016, we identified 30 cases of newborn haemorrhagic syndromes on 594 hospitalizations (5.05%). The sex-ratio (M/F) was 1.5. None of them received vitamin K after birth and all were breastfed. Preterm infants accounted for 10%. The presentation of haemorrhage encountered was dominated by visceral bleeding especially digestive (80%), followed by epistaxis (10%), Haematuria (7%), and skin haemorrhage (3%). Physical examination was normal in most of cases with exception (nine babies had pallor with hypotonia, three babies suffered from hypovolemic shock, respiratory distress(10%), drowsiness, poor sucking and fever. The most common cause of bleeding disorder was haemorrhagic disease of the new-born (80%), disseminated intravascular coagulation (DIC) (10%), esophagitis (6.67%) and isolated thrombocytopenia (3.33%). At the end of our study, given the high frequency of vitamin k deficiency bleeding disease despite the prophylaxis received, a strengthening of the prevention system is necessary.

## Introduction

The haemorrhagic disorders are particularly frequent in neonatal period. Causes of bleeding in the neonate are multifactorial, and include disseminated intravascular coagulation, Vitamin K deficiency, hereditary bleeding disorders, thrombocytopenia, platelet function defects, hepatic disease, and trauma [1]. In 1894 Townsend [2] first used spontaneous the term “haemorrhagic disease of the newborn” to describe the spontaneous bleeding' occasionally encountered in newborn infants, and he reported fifty cases from the Bosten Lying-in Hospital. He was unable to determine the cause for the condition but recognized that he was dealing with a definite clinical syndrome resulting from a deficiency in Vitamin K and Vitamin K-dependent cofactors. Haemorrhagic disease of the new-born (HDN) is a rare disease with high mortality and morbidity [3]. It is one of the most frequent causes of intracranial haemorrhage in the first year of life. New-borns have only 20-50% of adult coagulation activity. Lack of vitamin K administration at birth, exclusive breast feeding, chronic diarrhea and prolonged use of antibiotics make them more prone to vitamin K deficiency bleeding [4]. Through this work, we tried to describe presenting clinical and laboratory features of new-born haemorrhagic disorders and determine their different causes.

## Methods

Between December 2015 and April 2016 thirty patients were admitted to paediatric emergency medical department of Rabat Children's Hospital, with the diagnosis of onset haemorrhagic disorder. The detailed history included the mother's general health and medical condition prior to delivery, the place of birth, and whether vitamin K was given at birth. Laboratory studies included complete blood count, partial thromboplastin time (PTT), prothrombin time (PT), and liver function tests. Vitamin K-dependent factor activities were performed when possible. After blood had been drawn for the baseline evaluation, vitamin K (l-2mg) was given intravenously. In babies with severe anaemia packed red blood cells and fresh frozen plasma were given. Cranial ultrasonography and computed tomography (CT) were performed in all babies for the diagnosis and the evaluation of intracranial bleeding. Follow-up of all infants was carried-out monthly in the child neurology unit for the first 3 months, and every 3 months thereafter. The outcome was assessed by head circumference, neurologic examination, cranial CT and/or magnetic resonance imaging (MRI), electroencephalographic study.

## Results

**Figure 1:**
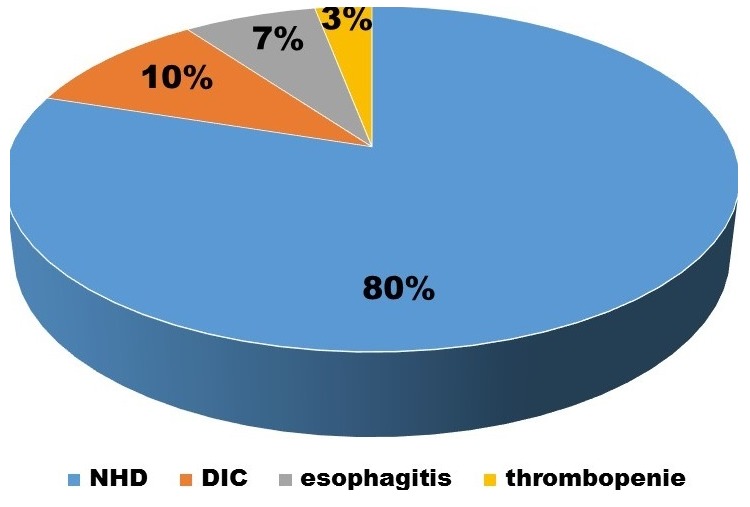
Causes of bleeding disorder

Our study encompassed eighteen males and twelve females. The mean gestational age of the population was 38 weeks. Five babies were post-term while three new-borns were preterm. Twenty-seven new-borns had normal spontaneous delivery. Two of the deliveries were at home. Twenty-five childs had been born at a local birthing center, attended solely by midwives. According to the mothers, there were “no problems” with the pregnancy or delivery, although the mother did not know the number of antenatal visits, extent of antenatal laboratory evaluation, or details of the infant's perinatal care. No further information could be obtained from the birthing center. The mean birth weight was 2700g. The delivery histories were uneventful and the family histories were negative for any form of hereditary or acquired bleeding disorder. None of them had received vitamin K at birth. Twenty-seven babies were fully breastfed; three babies were on mixed feeding. The presenting complaints and examination findings are described in Table 1. There were no histories of antibiotic usage, protracted diarrhoea in all babies. The laboratory evaluation revealed the following: the meanhaemoglobin 16 g/dl, nine babies presented anaemia, while four babies had thrombocytopenia. Three babies had leukopenia. The PT and PTT values of twenty-seven babies were longer than control values. In three patients, PT was longer than 60 seconds and MT longer than 120 seconds. Fibrinogen levels was low in three cases. Liver function tests were normal in twenty-seven cases. Vitamin K dependent factor activities (factors II, VII, IX and X) measured in three infants were decreased. Cranial and abdominal ultrasounds were normal in all our cases. The most common cause of bleeding was Haemorrhagic disease of the newborn, a disease resulting from a deficiency in Vitamin K and Vitamin K-dependent cofactors, it was seen in twenty-four babies (80%), followed by Disseminated intravascular coagulation (DIC) in three cases (10%), esophagitis in two cases and isolated thrombocytopenia in one cases (Figure 1). Bleeding tendency was treated in all patients with 1-2 mg vitamin K2 intravenously. Blood, platelet and fresh-frozen plasma (FFP) transfusions with curative antibiotic in DIC. Proton pump inhibitor (PPI) was administered in patients with esophagitis and platelet transfusion in case of isolated neonatal thrombocytopenia.

**Table 1:** Characteristics and clinical data at the admission

	Number of cases	Percentage
**Presentation of Haemorrhage**		
Digestive bleeding	24	80%
epistaxis	3	10%
Haematuria	2	7%
cutaneous haemorrhages	1	3%
*Physical examination		
Normal	21	70%
Hypovolemic shock	3	10%
Pallor	9	30%
Hypotonia	9	30%
Respiratory distress	3	10%

* More than one finding could be present in one patient.

## Discussion

New-born Haemorrhagic syndromes are frequent, and represent 2.17% of neonatal hospitalization in Salem et al. [5] and 5.9% in Jabnoun et al. [6]. There are many causes of bleeding disorder in the neonate and include disseminated intravascular coagulation, Vitamin K deficiency, hereditary bleeding disorders, thrombocytopenia, platelet function defects, hepatic disease, and trauma [1] (Table 2). Haemorrhagic disease of the newborn (HDN) is one of the most frequent bleeding disorders in infancy [4]. First described by Townsend, [2] results from a deficiency in Vitamin K and, consequently, in the coagulation factors dependent on Vitamin K. In umbilical blood the level of factors II, VII, IX, and X are nearly normal, but then decline to a nadir usually between 48 and 72 hours after birth. Levels slowly rise as dietary supplies of Vitamin K and establishment of intestinal flora allow hepatic synthesis of dependent cofactors s but remain below adult values for the first several weeks of life [7]. Cow's milk contains 60μg/L of Vitamin K; breast milk contains 15 μg/L [8,9]. Consequently, symptomatic haemorrhage (occurring in 0.25% to 0.5% of un-prophylactically treated infants [10] has been noted more frequently in breast-fed than in formula-fed infants unless Vitamin K prophylaxis is given [7,9]. Vitamin K deficiency can also occur due to secondary causes. Chronic diarrhea, cystic fibrosis, biliary atresia, celiac disease, alpha 1-antityripsin deficiency, abetalipoproteinemia and a history of war far in usage for a long period may induce vitamin K deficiency [11]. In our study, none of our patients had received vitamin K at birth and there was no history of antibiotic usage, protracted diarrhoea. The usual presentation is a manifestation of haemorrhage; melena, umbilical bleeding, haematuria, epistaxis, generalized ecchymosis, and circumcision site bleeding are possible manifestations, and the neonate also may bleed from iatrogenic puncture sites [12]. Physical examination usually is within normal limits except for the manifestation of bleeding unless the disease is complicated by intracranial haemorrhage or hypovolemic shock. Laboratory evaluation reveals prolongation of both the PT and PTT, reflecting Factors II, VII, IX, and X deficiencies, but the platelet count, peripheral smear, fibrinogen level, thrombin time, and fibrin split products are normal [12].

**Table 2:** Most common causes of bleeding in the neonate

**Disseminated intravascular coagulation**	Complications of pregnancy (abruption, previa, dead twin, maternal shock) Hypoxia Septicemia (bacterial - - group B strep) (viral - - TORCH) Respiratory distress syndrome. Necrotizing enterocolitis
**hemorrhagic disease of the newborn**	Vitamin K deficiency
**Hereditary bleeding disorders Clotting factors**	Hemophilia A and B Other factor deficiencies. AfibrinogenemiaJdysfibrinogenemia Platelets von Willebrand's Hereditary and familial thrombocytopenia Bernard Soulier Syndrome Wiscott Aldrich
**Thrombocytopenia**	Infections (sepsis, viremia)Intravascular coagulation syndromes s/p exchange transfusion Immune disorders (SLE, ITP, antiplatelet antibodies)
**Platelet function defects**	Uremia Acidosis Sepsis
**Hepatic disease**	Hepatitis (TORCH) Alpha-l-antitrypsin deficiency Metabolic defects
**Trauma**	Child abuse if unexplained

The incidence of vitamin K deficiency bleeding in early infancy was calculated to be decreased in Germany from l/14 000 to l/70 000 with single oral prophylaxis and to l/420 000 with single parenteral vitamin K prophylaxis [13]. The occurrence of late HDN in Sweden was l/19 570 among new-borns who had received vitamin K orally at birth [14]. Three patterns of haemorrhages due to vitamin K deficiency in infancy are identified. Early haemorrhagic disease of the new-born (HDN) occurs at birth or within the first 24 hours of delivery, frequently seen in babies whose mothers are on antitubercular (isoniazid and/or rifampicin), or antiepileptic (such as phenytoin and phenobarbital) drugs. It is often life threatening [4]. Classical HDN accounts for gastrointestinal, nasal, skin and circumcision bleeding that occurs between 2-5 days of neonatal period and life-threatening bleeding is rare. Late-onset disease is almost exclusively confined to breastfed infantsand can be seen during infancy but predominantly at 4-8 weeks of life.Late HDN can present with convulsions, poor sucking, irritability and pallor. Haemorrhages of gastrointestinal system, mucosal membranes and skin can accompany the disease.Mortality is reported in 14-50% cases by various authors [15]. Risk of intracranial haemorrhage in late HDN is reported in 50-80% cases [16]. While subdural is the most common location for haemorrhage, subarachnoid haemorrhage is the second most common type. Administering vitamin K to every new-born at birth can impede the disease, which has a high morbidity and mortality [11,15]. Oral prophylaxis of vitamin K is preventive against early and classical haemorrhagic disease, but parenteral administration of vitamin K is required for the late disease [13,16]. Current recommendations for vitamin K prophylaxis are to give vitamin K to all new-borns as a single intramuscular dose of 0.5 to 1 mg. Findings of Cochrane review (2009) are as follows - “A single dose (1.0 mg) of intramuscular vitamin K after birth is effective in the prevention of classic HDN. Either intramuscular or oral (1.0 mg) vitamin K prophylaxis improves biochemical indices of coagulation status at one to seven days. Neither intramuscular nor oral vitamin K has been tested in randomized trials with respect to effect on late HDN. If intracranial or other serious haemorrhage occurs administration, either IM or IV, is both safe and effective, an infusion of 10 to 15 mL/kg of fresh frozen plasma will immediately correct the haemostatic defect and clinical haemorrhage usually stops within two hours of administration. This infusion also is used if the coagulation defect is unknown because haemostatic levels (> 20%) of all potential factor deficiencies (except congenital hypoprothrombinaemia and afibrinogenemia) and cessation of bleeding will result [1]. Profoundanaemia and shock may be corrected by 20 mL/kg whole blood transfusion.

**In our study:** Newborn haemorrhagic disease was the most frequent bleeding disorder, it was seen in twenty-four babies (80%), and the early form concerned seven babies while the classic form was found in seventeen cases. No late forms were observed in our series. The PT and PTT were longer than control values. Vitamin K was given IM, and 10 mL/kg of fresh frozen plasma was administered over two hours. Following this therapy, the bleeding stopped within the next two hours, and no further bleeding sites developed. Disseminated intravascular coagulation (DIC) was noted in 3 cases, they had hypovolemic shock: they were lethargic, very pale with profound hypotonia, tachycardia, tachypnoea and oliguria at the presentation, the capillary refill time (CRT) was > 3 s. Laboratory evaluation reveals: pancytopenia, Prothrombin time (PT) was very low whereas partial thromboplastin time (PTT) was markedly elevated aggravated by renal and liver failure. Unfortunately, they died despite of Blood, platelet and fresh-frozen plasma (FFP) transfusions with curative antibiotic. Esophagitis has been noticed in 2 new-borns presented an isolated digestive haemorrhage with normal haemostasis. Isolated thrombocytopenia was observed in one case.

## Conclusion

The low concentration of vitamin K in human breast milk and the predisposition to vitamin K deficiency bleeding following exclusive breast feeding is emerging as a matter of concern especially in developing countries where exclusive breast feeding is vigorously advocated to promote optimal health in the infant. Most reports of late HDN have been in babies born at home and not given vitamin K prophylaxis.

### What is known about this topic

- Haemorrhagic disease of the new-born, a disease resulting from a deficiency in Vitamin K and Vitamin K-dependent cofactors, is a common presentation to the Emergency Department, must be considered in infants whose births were lay attended or if no definite history of Vitamin K administration to the infant can be obtained;
- Intracranial haemorrhage is the major cause of morbidity and mortality.

### What this study adds

- This study once again emphasizes the value of vitamin K prophylaxis in the new-born to reduce the incidence of onset haemorrhage and sequel in children;
- Therefore, strengthening of the prevention system is necessary.

## Competing interests

The authors declare no competing of interests.
